# Dynamic Fixation versus Static Screw Fixation for Syndesmosis Injuries in Pronation External Rotation Ankle Fractures: A Retrospective Case Control Study

**DOI:** 10.5704/MOJ.2311.008

**Published:** 2023-11

**Authors:** CM Lim, SW Choi, BS Kim, SJ Lee, HS Kang

**Affiliations:** Department of Orthopaedic Surgery, Jeju National University Hospital, Jeju, South Korea

**Keywords:** ankle, syndesmosis injury, dynamic fixation, screw fixation

## Abstract

**Introduction:**

The current standard treatment for ankle syndesmosis injury is static screw fixation. Dynamic fixation was developed to restore the dynamic function of the syndesmosis. The purpose of this study was to determine that which of static screw fixation and dynamic fixation is better for treatment of ankle syndesmosis injury in pronation-external rotation fractures.

**Materials and methods:**

Thirty patients were treated with dynamic fixation (DF group) and 28 patients with static screw fixation (SF group). The primary outcome was Olerud–Molander Ankle Outcome Score. The secondary outcome were Visual Analogue Scale score and American Orthopedic Foot and Ankle Society score, radiographic outcomes, complications and cost effectiveness. To evaluate the radiographic outcome, the tibiofibular clear space, tibiofibular overlap, and medial clear space were compared using the pre-operative and last follow-up plain radiographs. To evaluate the cost effectiveness, the total hospital cost was compared between the two groups

**Results:**

There was no significant difference in primary outcome. Moreover, there were no significant difference in secondary outcome including Visual Analogue Scale score and American Orthopedic Foot and Ankle Society score and radiographic outcome. Two cases of reduction loss and four cases of screw breakage were observed in the SF group. No complication in the DF group was observed. Dynamic fixation was more cost effective than static screw fixation with respect to the total hospital cost.

**Conclusion:**

Although dynamic fixation provided similar clinical and radiologic outcome, dynamic fixation is more cost effective with fewer complications than static screw fixation in ankle syndesmosis injury of pronation-external rotation fractures.

## Introduction

Ankle syndesmosis injury may occur in 10% – 15% of ankle fractures, and approximately 20% of ankle fractures require surgical stabilisation^1-3^. Any injuries of the ankle mortise may lead to a significant ankle joint dysfunction. Some mechanical studies have demonstrated that a 1mm lateral shift of the talus in the ankle mortise results in a 40% loss of tibiotalar contact surface area and a 43% increase in ankle volume^4,5^. A persistent unstable syndesmosis injury has the potential to develop into chronic ankle pain, early degenerative change, functional disabilities, instability, and latent diastasis^6,7^.

The current standard treatment for ankle syndesmosis injury is static screw fixation with one or multiple screws of different sizes through three or four cortices^8^. Despite adequate reduction and stable fixation, static screw fixation does not uniformly have excellent outcomes, and one possible reason for poor results is non-anatomic reduction^9^. Gardner *et al* reported a 52% incidence of malreduction of the tibiofibular syndesmosis in Weber C ankle fractures treated with screw fixation^10^. Even when the reduction is anatomic, screw fixation has potential complications. Rigid screw fixation eliminates the dynamic properties of the syndesmosis, which could lead to pain, decreased motion, fixation loosening, reduction loss, or screw breakage. Routine screw removal to avoid hardware failure necessitates exposing the patient to a second operation^9^.

Dynamic fixation using TightRope system [Arthrex, Naples, FL] was developed to restore the dynamic function of the syndesmosis while maintaining the reduction^11^. Numerous studies have confirmed that dynamic fixation allows micromotion of the syndesmosis with reduction and longer stability, which in turn facilitates early mobility, weight bearing, and faster return to work^12,13^. Furthermore, dynamic fixation is recommended for osteoporosis, whereas screw fixation may lead to cut-out due to low fixation strength^14,15^. Another potential advantage of dynamic fixation is the avoidance of implant removal^14^. Nevertheless, a loose dynamic fixation may lead to subsequent syndesmosis diastasis^16^, and some complications, including infection and pain over the knot, have been reported^13,17^.

There are many studies for evaluating the efficacy between dynamic fixation and static screw fixation in ankle syndesmosis injury. However, most of these studies did not consider the type of ankle fracture accompanying syndesmosis injury. There were some studies proved that the syndesmotic fixation did not influence the functional and radiologic outcome in supination-external rotation fractures (Weber B). Therefore, we included only pronation-external rotation fractures (Weber C) in this study.

The purpose of this retrospective case control study was to determine that which of static screw fixation and dynamic fixation is better for treatment of ankle syndesmosis injury in pronation-external rotation fractures (Weber C). For this we compared the clinical and radiographic outcomes and the cost effectiveness between dynamic fixation and static screw fixation. We hypothesise that the dynamic fixation technique provides better clinical outcomes and fewer complications and is more cost effective than static screw fixation for ankle syndesmosis injury in pronation-external rotation fractures (Weber C).

## Materials and Methods

This study was approved by the appropriate Institutional Review Board (Ethics Committee, IRB number: 2020-12014, date of approval: January 15th, 2021). All methods were performed in accordance with the relevant guidelines and regulations (Declaration of Helsinki). The requirement for informed consent was waived by the Institutional Review Board because of the retrospective nature of the study.

We performed a retrospective case control study from January 2017 to December 2019, on 69 patients with Lauge– Hansen pronation-external rotation (PER) type / Weber C ankle fracture with syndesmosis injury. Between 2017 and 2018, we performed static screw fixation for ankle syndesmosis injury. Between 2018 and 2019, dynamic fixation was preferred due to its advantages reported in the literature. The inclusion criteria were as follows: (1) age between 18 and 65 years; (2) Lauge–Hansen PER type / Weber C ankle fracture; (3) pre-operative and intra-operative acute syndesmosis injury based on plain radiographs (tibiofibular clear space >6mm, tibiofibular overlap <6mm, and medial clear space <4mm) and the intra-operative cotton test (tibiofibular clear space >5mm); (4) duration of <7 days from trauma to operation. The patients who had polytrauma or open fracture (n=3), neurologic impairment (n=1), obesity (body mass index ≥40) (n=2), or follow-up periods of <2 years (n=5) were excluded.

**Fig 1:**
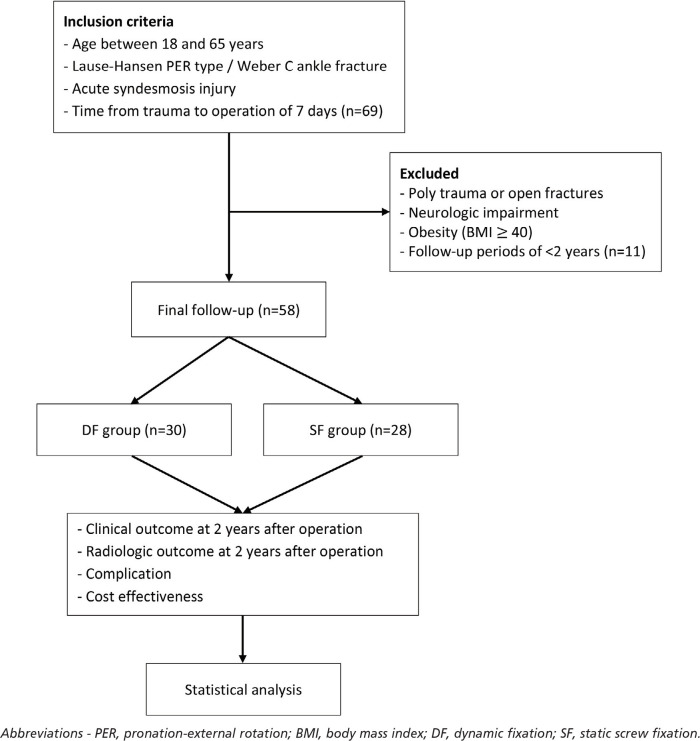
Flowchart of patient inclusion and study.

We enrolled 58 patients with Lauge–Hansen PER type / Weber C ankle fracture with syndesmosis injury. Among them, 30 patients each were treated with dynamic fixation (DF group) and 28 patients with static screw fixation (SF group) for acute syndesmosis injury (Fig. 1). This patient size satisfied the optimal sample size which was calculated by G-Power program (a free statistical program available at http://www.gpower.hhu.de/). The significance level (α), statistical power (1-ß), and effect size (f) were set at 0.05, 0.8, and 0.5, respectively.

Dynamic fixation and static screw fixation techniques were almost similar. Most surgeries were performed under spinal anaesthesia in both the groups and within seven days from trauma. Associated lateral, medial, and/or posterior malleolus was treated with open reduction and internal fixation according to standard principles before stabilising the syndesmosis. In Maisonneuve fractures, also known as proximal fibula fractures, no fixation was performed. Temporary syndesmosis reduction was obtained in both the groups by direct syndesmosis compression with large reduction forceps. Stability was confirmed using fluoroscopy. No routine open reduction or debridement of the syndesmosis space was performed.

TightRope system [Arthrex, Naples, FL] was used for dynamic fixation. Under fluoroscopic guidance, a 3.5mm drill hole was made approximately 2cm above and parallel to the distal tibia joint line from 30° posterior to anterior up to the lateral tibial cortex through a hole in the plate, if present. A guide needle was inserted from the lateral to medial side through the drill hole to bring the endobutton over the medial cortex of the tibia. The endobutton was flipped by releasing pressure on the needle medially and pulling the fibre wire suture laterally. Fixation was achieved with a surgical knot. We made sure that the endobutton lay flat on the medial cortex of the tibia without any soft tissue interposition to prevent subsequent loosening (Fig. 1). After achieving stable fixation by either technique, the reduction forceps were removed, and stability was assessed under fluoroscopy.

**Fig 2:**
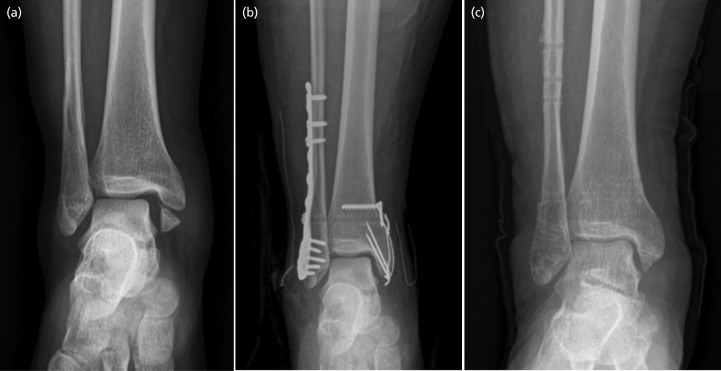
Dynamic fixation of syndesmosis. (a) Pre-operative anteroposterior radiograph of right ankle showing pronation external rotation-type ankle fracture. (b) The syndesmosis was fixed with TightRope. (c) At the one-year follow-up, all implants were removed; radiograph showing complete bone union and well-maintained ankle mortise.

Under fluoroscopic guidance, a 2.5mm drill hole was made approximately 2cm above and parallel to the distal tibia joint line from 30° posterior to anterior up to the lateral tibial cortex through a hole in the plate if present. Three or four cortices were drilled, and a 3.5mm cortical screw was inserted (Fig. 2). The static screw was routinely removed eight weeks after the operation.

Post-operative management was the same for both the groups. Posterior short-leg splints were applied for the first two weeks. Thereafter, the splint was removed, and below-knee, short-leg cast was applied for four more weeks. Weight bearing was not allowed during this period. At eight weeks, weight bearing ankle anteroposterior, mortise, and lateral radiographs were obtained. If the syndesmosis appeared stable and any associated fractures were healed, the cast was removed and progressive rehabilitation (progressive weight bearing and ankle range of motion exercise) was allowed. Patients had an outpatients visit for the first two weeks, four weeks, three months and six months after operation, and then every six months thereafter. The Clinical and radiologic outcomes were analysed based on the results two years after operation.

The primary outcome was the clinical outcome based on Olerud–Molander Ankle Outcome Score (OMAS). OMAS has been validated in patients treated for ankle fracture and consists of nine criteria grouped into nine parameters: the maximum score is 100 points.

The secondary outcome were the clinical outcomes including Visual Analogue Scale (VAS) score and American Orthopedic Foot and Ankle Society (AOFAS), radiographic outcome, cost effectiveness and potential complications. Ankle pain was evaluated using VAS score. AOFAS score is widely used by surgeons for ankle and hindfoot problems, which consists of nine criteria grouped into three categories: pain (1 criterion, 40 points), function (7 criteria, 50 points), and alignment (1 criterion, 10 points); the maximum score is 100 points. Clinical evaluation was performed at two years post-operatively.

The radiographic outcome was measured with pre-operative and post-operative plain radiographs (anteroposterior view). The tibiofibular clear space (TFCS), tibiofibular overlap (TFO), and medial clear space (MCS) were compared between the two groups to evaluate the adequate reduction and loss of reduction. Post-operative radiographic outcome was measured at the final follow-up (two years postoperatively).

To evaluate the cost effectiveness between both the groups, the total hospital cost associated with ankle fracture and syndesmosis injury were compared. In this study, the static screw was routinely removed eight weeks after the operation and all implants were routinely removed approximately one year after the operation. The total hospital cost of the DF group consisted of the initial admission cost for ankle fracture with syndesmosis injury and the final admission cost for all implant removal. The total hospital cost of the SF group consisted of the initial admission cost for ankle fracture with syndesmosis injury, the second admission cost for static screw removal, and the final admission cost for all implant removal. In the presence of complications, the admission cost for the complications was included into the initial admission fee.

Potential complications of syndesmosis fixation, such as, malreduction, implant failure, soft tissue irritation, infection, syndesmosis ossification, or osteolytic reaction were evaluated.

Inter-observer and intra-observer reliabilities were assessed using intraclass correlation coefficient of the radiographic measurements, and an agreement of 0.75 was considered excellent. To determine significant differences in VAS, OMAS, and AOFAS scores and the radiographic parameters between the two groups, Student’s t-test was performed. A p-value ≤0.05 was considered statistically significant. Data were analysed using Statistical Package for the Social Sciences 19.1 software [IBM, Chicago, IL, USA].

## Results

There was no significant difference in baseline characteristics such as, age at operation, BMI, smoking, diabetes, working type, mechanism of injury and follow-up periods by both groups (Table I).

**Table I:** Demographics. There was no significant difference in baseline characteristics between two groups

	DF group (N = 30)	SF group (N = 28)	p
Age at operation, y*	35.4 ± 15.4	34.9 ± 13.7	0.910
Gender			
Male	20 (67%)	19 (68%)	
Female	10 (33%)	9 (32%)	
BMI	26.8±4.3	27.5±2.3	0.443
Smoking			
Yes	8 (27%)	8 (29%)	1.000
No	22 (73%)	20 (71%)	
Diabetes			
Yes	1 (3%)	1 (4%)	0.945
No	29 (97%)	27 (96%)	
Working type			
Mainly physical	20 (76%)	21 (75%)	0.657
Mainly intellectual	10 (33%)	7 (25%)	
Mechanism of injury			
Low energy fall	19 (63%)	18 (29%)	0.732
Sports	7 (23%)	7 (25%)	
Motor vehicle injury	4 (13%)	2 (7%)	
Fracture characteristics			
Maisonnneuve	2	1	
Fibular only	4	3	
Fibular and medial malleolus	9	7	
Fibular and posterior malleolus	6	9	
Trimalleolar fracture	9	8	
Follow-up period, y*	2.4 ± 0.3	2.7 ± 0.7	0.059

Notes: *Results expressed as mean (SD, standard deviation), BMI (body mass index)

There was no significant difference in primary outcome. The average OMAS scores in the DF group and SF group were 95.2±7.0 (range, 80 – 100) and 90.5±7.5 (range, 80 – 100), respectively. Moreover, there were no significant difference in secondary outcome including Visual Analogue Scale (VAS) score and American Orthopedic Foot and Ankle Society (AOFAS), radiographic outcome. The average VAS scores in the DF group and SF group were 0.7±1.1 (range, 0 – 4) and 1.1±1.2 (range, 0 – 4), respectively. The average AOFAS scores in the DF group and SF group were 93.5±7.9 (range, 78 – 100) and 88.0±5.9 (range, 78 – 100), respectively. There was no significant difference in the VAS (p=0.058), OMAS (p=0.058), and AOFAS scores (p=0.058) between both the groups (Table II).

**Table II:** Clinical outcomes. There was no significant difference in clinical outcomes between two groups

	DF group	SF group	p
VAS	0.7 ± 1.1	1.1 ± 1.2	0.73
OMAS	95.2 ± 7.0	90.5 ± 7.5	0.78
AOFAS score	93.5 ± 7.9	88.0 ± 5.9	0.85

Note: Results expressed as mean ± standard deviation. Abbreviation - VAS: visual analogue scale, OMAS: Olerud-Molander Ankle Outcome Score, AOFAS: American Orthopedic Foot and Ankle Society

Each radiographic measurement showed good to excellent interobserver and intra-observer agreement. Adequate syndesmosis reduction was achieved after surgery in both the groups. In the DF group, the average TFCS was 7.1±1.8 mm (range, 5.3 – 12.0mm) before operation and 4.5±0.8mm (range, 3.0 – 6.8mm) at the final follow-up. The average TFO was 1.9±1.3mm (range, 0 – 4.3mm) before operation and 6.3±1.7mm (range, 4.2 – 8.8mm) at the final follow-up. The average MCS was 7.6±2.7mm (range, 5.5 – 12.9mm) before operation and 2.7±0.6mm (range, 1.5 – 3.6mm) at the final follow-up. In SF group, the average TFCS was 7.1±2.2mm (range, 5.9 – 11.3mm) before operation and 4.8±0.9mm (range, 3.5 – 6.8mm) at the final follow-up. The average TFO was 1.9±1.1mm (range, 0 – 3.2mm) before operation and 6.2±2.0 mm (range, 3.2 – 8.9mm) at the final follow-up. The average MCS was 7.7±1.8mm (range, 4.3 – 11.9mm) before operation and 2.9±0.6mm (range, 1.7 – 3.7mm) at the final follow-up. The TFCS, TFO, and MCS significantly improved after surgery in both the groups. Adequate syndesmosis reduction was achieved in both the groups, and no significant difference between the two groups was found (Table III).

**Table III:** Radiologic outcomes. There was no significant difference in radiologic outcomes between two groups

	DF group	Pre-operative SF group	p	DF group	Final follow-up SF group	p
TFCS (mm)	7.1 ± 1.8	7.1 ± 2.2	0.831	4.5 ± 0.8	4.8 ± 0.9	0.116
TFO (mm)	1.9 ± 1.3	1.9 ± 1.1	0.106	6.3 ± 1.7	6.2 ± 2.0	0.848
MCS (mm)	7.6 ± 2.7	7.7 ± 1.8	0.575	2.7 ± 0.6	2.9 ± 0.6	0.399

Note: Results expressed as mean ± standard deviation. Abbreviations - TFCS: tibiofibular clear space, TFO: tibiofibular overlap, MCS: medial clear space

Dynamic fixation was more cost effective than static screw fixation with respect to total hospital cost for ankle syndesmosis injury. The average total hospital cost in the DF group and SF group was 2,172,013±343,695 ₩ (Korea Won, KRW) and 2,437,469±324,445 ₩, respectively. The average total hospital cost of the DF group was significantly lower than that of the SF group (p=0.058). The cost of TightRope for dynamic fixation was 132,988 ₩ and the cost of cortical screw for static screw fixation was 19,383 ₩. Despite the difference of cost between TightRope and cortical screw, the potential cost saving was about 250,000 ₩ per case, which is equivalent to the cost of the second surgery for screw removal in the SF group (Table IV).

**Table IV:** Cost effectiveness. Dynamic fixation was more cost effective than static screw fixation.

	DF group	SF group	p
Initial admission cost (₩)	1,645,891 ± 336,095	1,447,274 ± 296,306	0.045
Second admission cost (₩)		453,720 ± 61,768	
Final admission cost (₩)	526,123 ± 61,958	536,476 ± 74,289	0.635
Total cost (₩)	2,172,013 ± 343,695	2,437,469 ± 324,445	0.016

Note: Results expressed as mean ± standard deviation.

**Fig 3:**
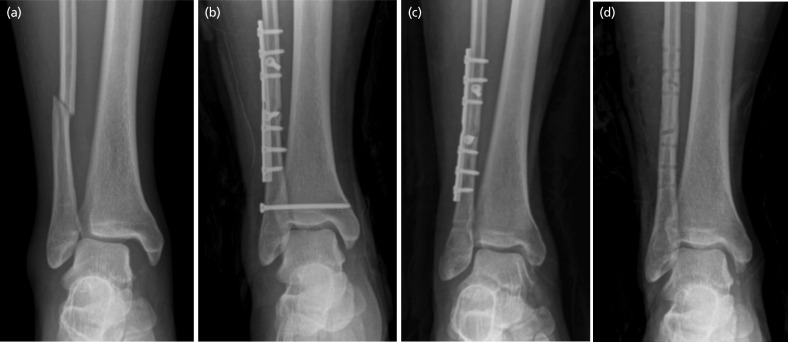
Static screw fixation of syndesmosis. (a) Pre-operative anteroposterior radiograph of right ankle showing supination external rotation-type ankle fracture. (b) The syndesmosis was fixed with a cortical screw. (c) At eight weeks post-operatively, the syndesmotic screw was removed. (d) At the one-year follow-up, all implants were removed; radiograph showing complete bone union and well-maintained ankle mortise.

In the SF group, six patients (20%) had complication. Among them, two patients required corrective surgery (screw removal and new screw positioning), and four patients had fixation failure, which was observed as a broken screw; of the four patients, one patient had a failed screw removal (Fig. 3). No failure was found in the DF group.

**Fig 4:**
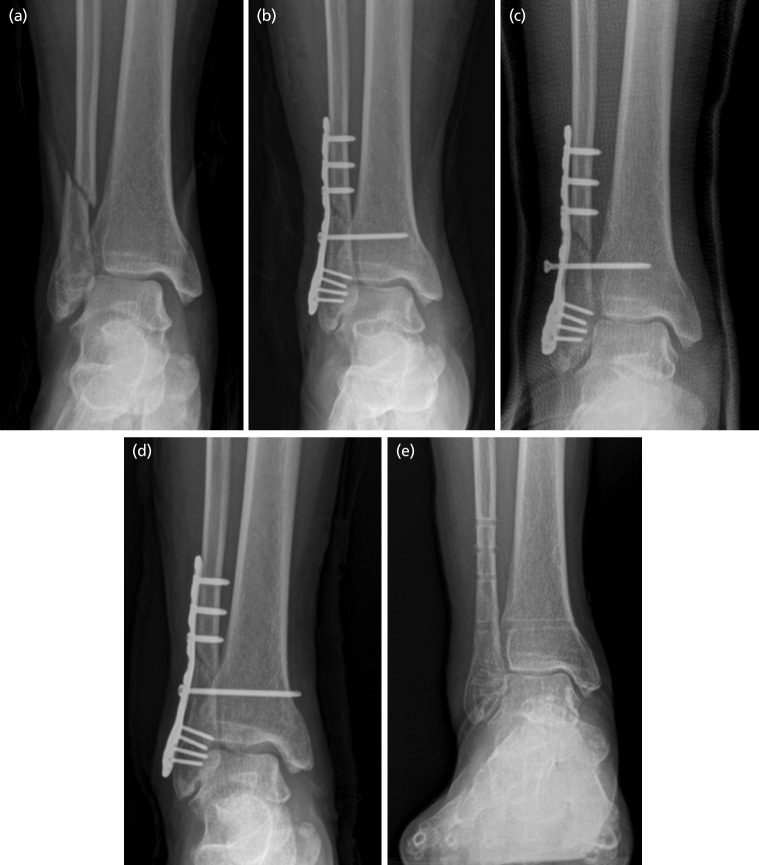
Complication of screw fixation. (a) Pre-operative anteroposterior radiograph of right ankle showing supination external rotation-type ankle fracture. (b) The syndesmosis was fixed with a cortical screw. (c) At four weeks post-operatively, the syndesmosis reduction was loss due to screw cut out. (d) The syndesmosis was re-fixed with a cortical screw. (e) At the one-year follow-up, all implants were removed; radiograph showing complete bone union and well-maintained ankle mortise.

## Discussion

The main finding of this study is that although dynamic fixation provided similar clinical and radiologic outcomes, it was more cost effective with fewer complications than static screw fixation in ankle syndesmosis injury of pronation-external rotation fractures (Weber C). Although the average OMAS and AOFAS scores of the DF group were higher than that of the SF group, there was no significant difference. Moreover, dynamic fixation was more cost effective with fewer complications than static screw fixation. As a result, dynamic fixation is more effective than static screw fixation in ankle syndesmosis injury of pronation-external rotation fractures (Weber C).

The current retrospective study demonstrated similar clinical outcomes between dynamic fixation and static screw fixation for ankle syndesmosis injury. There was no significant difference in the primary outcome (OMAS) and secondary outcome including VAS score and AOFAS. However, there is an ongoing debate about the differences in clinical outcomes between the two fixation methods. Raeder *et al* reported that the average OMAS and AOFAS scores of dynamic fixations were significantly higher than those of static screw fixation^3^. However, the difference between OMAS scores was below minimal clinically significant differences (MCID) and the MCID for AOFAS scores is unknown for ankle fractures. Therefore, it is unclear whether these differences were clinically significant. Laflamme *et al* also reported better clinical results in those undergoing the dynamic fixation operation. In their study, the OMAS score was 93.3±10.2 with dynamic fixation and 87.7±12.2 with static screw fixation (p=0.046)^14^. However, this difference was below the MCID for OMAS score (MCID for OMAS is 12 points). Thus, the difference described by Laflamme *et al* was not clinically significant. Lehtola *et al* reported better clinical results in static screw fixation in terms of OMAS. However, the difference between the mean values was not statistically significant and below the MCID for OMAS^18^. As a result, dynamic fixation provides clinical outcomes equivalent to those of static screw fixation.

Although similar clinical outcomes between dynamic fixation and static screw fixation were reported, many studies have reported that the potential advantage of the dynamic nature of dynamic fixation may allow for physiologic micromotion of syndesmosis. Static screw fixation did not allow weight bearing before the screw removal (i.e., till eight weeks after the operation) as weight bearing might lead to screw breakage or loosening. However, dynamic fixation allows the patients to return earlier to weight bearing without reduction loss or implant failure^19^. Degroot *et al* reported an average of 5.7 weeks of full weight bearing time with dynamic fixation, without residual displacement^13^. Thornes *et al* showed that the dynamic fixation group had a shorter average time of weight bearing than the static screw fixation group (4.1 weeks versus 6.3 weeks, p=0.01)^16^. Moreover, some studies have demonstrated that early weight bearing could accelerate rehabilitation and early return to work^12,16^. The potential advantage of dynamic fixation is that it permits micromovement of the distal tibiofibular joint and normal rotation during the normal gait cycle, which in turn results in better clinical outcomes in terms of mobility, accelerated ligament healing, early return to full weight bearing, and return of physical abilities^17^. In this study, in order to compare dynamic fixation and static screw fixation, we did not allow the patients of the two groups to bear weight for eight weeks. However, we are also in agreement that one should allow for early full weight bearing in dynamic fixation.

Malreduction of the ankle syndesmosis can lead to mortise widening and further osteoarthritis^20^. In this study, the radiographic outcomes (average TFCS, TFO, and MCS) improved significantly and adequate syndesmosis reduction was achieved post-operatively in both the groups. Although all patients had adequate reduction, one patient in the SF group required corrective surgery (screw removal and new screw positioning). Many previous studies have reported that there was no malreduction in dynamic fixation. Naqvi *et al* compared the accuracy of reduction between static screw fixation and dynamic fixation using computed tomography (CT) and reported that dynamic fixation provides more accurate reduction than screw fixation^21^. Lafalmme *et al* have performed a prospective randomised multicenter trial to compare the post-operative clinical and radiographic outcomes between static screw fixation and dynamic fixation. Loss of reduction was observed in four patients, of which three occurred after screw removal in the screw fixation group (36 patients)^14^. Zhang *et al* reported 1% (1 of 93 patients) of malreduction in dynamic fixation and 12.6% (12 of 95 patients) in static screw fixation in their systematic review study^19^. Rigby *et al* have demonstrated that the dynamic nature of dynamic fixation allows some degree of physiologic micromotion of the syndesmosis, thereby leading to syndesmosis reduction^22^. In terms of radiologic results, the dynamic fixation demonstrated more accurately reduced results and maintained the ankle syndesmosis than the screw fixation group.

There are many studies for evaluating the efficacy between dynamic fixation and static screw fixation in ankle syndesmosis injury. However, most of these studies did not consider the type of ankle fracture accompanying syndesmosis injury. There were some studies proved that the syndesmotic fixation did not influence the functional and radiologic outcome in supination-external rotation fractures (Weber B). Pakarinen *et al* performed a prospective randomised study to evaluate whether transfixation of syndesmosis injury is necessary in supination-external rotation fractures (Weber B)^23^. They reported that syndesmosis fixation did not influence the functional outcome in supination-external rotation fractures. Moreover, Lehtola *et al*, reported long term results of a prospective randomised study to evaluate the clinical relevance of syndesmosis fixation in supination-external rotation ankle fractures. They concluded that supination-external rotation ankle fractures with unstable syndesmosis can be treated with only malleolar fixation with good to excellent long term functional outcomes^24^. In this study, we included only pronation-external rotation fractures (Weber C). Therefore, this study could more accurately evaluate the efficacy of synsdesmosis fixation.

In this study, we demonstrated that the dynamic fixation was more cost effective, and the average cost saving was about 250,000 ₩ per case, which is equivalent to the cost of the second surgery for screw removal in the SF group. Many previous studies have already reported the cost effectiveness of dynamic fixation. Schepers *et al* reported that the additional costs of static screw removal are around 700 Euro^7^. Inge *et al* reported that the cost saving of dynamic fixation with TightRope was $651.50 AUD per case which was based on a second operation for screw removal^25^. Although, it is difficult to compare cost effectiveness because each country has different medical insurance systems, this study will be helpful in comparing cost effectiveness in Korea with other countries. Moreover, regarding the cost effectiveness aspect, potential complications, number of follow-up clinic appointments, and time to return to work should be taken into consideration. Many studies have already reported lower risk of implant failure by using dynamic fixation and the patients in the DF group returned earlier to their previous working, which theoretically means lower medical costs^14,16^. As a result, dynamic fixation was more cost effective than static fixation.

Potential complications of syndesmosis fixation include implant failure, soft tissue irritation, infection, syndesmosis ossification, or osteolytic reaction^26^. In this study, we found two patients required corrective surgery (screw removal and new screw positioning) and four patients had screw breakage in the SF group. No complication was found in the DF group. One previous study has reported implant failure in static screw fixation. Laflamme *et al* reported three cases of reduction loss among 36 patients treated with static screw fixation^14^. Coetzee *et al* reported one case of screw breakage among 12 patients treated with static screw fixation^9^. Schepers *et al* demonstrated a 22% complication rate of static screw fixation; three cases of screw breakage and one case of reduction loss^27^. Static screw fixation does not allow physiologic motion of the syndesmosis during healing, it may cause the screw breakage and loosening^19^. However, Zhang *et al* reported that there was no implant failure in dynamic fixation in their systematic review study^19^. The soft tissue irritation and discomfort were the main complications of dynamic fixation. Rigby *et al* reported that seven patients had soft tissue irritation among 37 patients treated with dynamic fixation^22^. McMurray *et al* treated 16 patients with dynamic fixation, of which two patients underwent TightRope removal due to superficial infection secondary to local subcutaneous knot irritation^28^. Naqvi *et al* proposed some strategies to avoid skin irritation due to TightRope: a posterior short knot and/or reaming the posterior aspect of the fibula^21^. Dynamic fixation could be a useful alternative treatment option with fewer complications for an ankle syndesmosis injury if we are to make an effort to reduce skin irritation.

Our study has some limitations. This study was a retrospective study, and the sample size was small. Thus, further prospective randomised multicentre trials are required. CT scan was not used to assess the reduction quality that was performed. Hence, further studies should include CT evaluation. In addition, the two-year follow-up period prevented the detection of long-term results and complications, such as degenerative arthritis, or osteochondral lesion.

## Conclusions

Dynamic fixation provided similar clinical and radiologic outcome in ankle syndesmosis injury of pronation-external rotation fractures (Weber C). However, dynamic fixation was more cost effective with fewer complications than static screw fixation. In conclusion, dynamic fixation is more cost effective than static screw fixation in ankle syndesmosis injury of pronation-external rotation fractures (Weber C).
